# The Italian Version of the Test Your Memory (TYM-I): A Tool to Detect Mild Cognitive Impairment in the Clinical Setting

**DOI:** 10.3389/fpsyg.2020.614920

**Published:** 2021-01-18

**Authors:** Maria Rosaria Barulli, Marco Piccininni, Andrea Brugnolo, Cinzia Musarò, Cristina Di Dio, Rosa Capozzo, Rosanna Tortelli, Ugo Lucca, Giancarlo Logroscino

**Affiliations:** ^1^Department of Clinical Research in Neurology, Center for Neurodegenerative Diseases and the Aging Brain, University of Bari “Aldo Moro”, “Pia Fondazione Cardinale G. Panico”, Tricase, Italy; ^2^Institute of Public Health, Charité – Universitätsmedizin Berlin, Berlin, Germany; ^3^Department of Neuroscience, Rehabilitation, Ophthalmology, Genetics, and Mother-Child health (DINOGMI), University of Genoa, Genova, Italy; ^4^Clinical Psychology Unit, Istituto di Ricovero e Cura a Carattere Scientifico (IRCCS) Ospedale Policlinico San Martino, Genoa, Italy; ^5^UCL Huntington’s Disease Centre, UCL Queen Square Institute of Neurology, University College London, London, United Kingdom; ^6^Laboratory of Geriatric Neuropsychiatry, Department of Neuroscience, Istituto di Ricerche Farmacologiche Mario Negri IRCCS, Milan, Italy; ^7^Department of Basic Medical Sciences, Neuroscience and Sense Organs, University of Bari “Aldo Moro”, Bari, Italy

**Keywords:** cognitive screening test, test your memory-Italian version, TYM, mild cognitive impairment, dementia

## Abstract

The Test Your Memory (TYM) is a brief self-administered, cognitive screening test, currently used in several settings. It requires minimal administrator supervision and the computation of the final test score takes approximately 2 min. We assessed the discrimination ability of the Italian version of the TYM (TYM-I) in detecting Mild Cognitive Impairment (MCI) in clinical setting. TYM-I was administered to 94 MCI patients and 134 healthy controls. The clinical diagnosis of MCI was considered as the gold standard. An extended formal neuropsychological test battery was used to define MCI subtypes. Receiver Operating Characteristic (ROC) analyses were conducted to find the optimal cut-off and measure discrimination ability of TYM-I in detecting MCI. TYM-I had a similar area under the curve (AUC = 0.85) point estimate as Mini Mental State Examination (MMSE) (AUC = 0.83). A TYM-I score lower or equal to 36 was found to be optimal cut off to detect MCI. The TYM-I showed the highest discrimination ability among individuals aged more than 70 and high educational level (AUC = 0.89). The amnestic MCI subtype patients, compared to non-amnestic MCI patients, had worse performance in recall, orientation and visuospatial abilities TYM-I subscores. The TYM-I is a valid screening test in detecting cognitive dysfunction, easily carried out in clinical practice. The TYM-I subscores may allow to identify amnestic and non-amnestic MCI subtypes.

## Introduction

The identification of individuals with cognitive impairment and dementia is important to guide clinical practice and has several implications for research including clinical trial recruitment and the development of dementia preventive strategies (Stephan et al., 2007; Brayne et al., 2011). Mild Cognitive Impairment (MCI) refers to a heterogeneous condition, intermediate between normal cognitive status and dementia, classified with different systems (Petersen et al., 1999; Petersen, 2004; Winblad et al., 2004; Matthews et al., 2009). Prevalence of MCI varies widely across different populations (between 0.1 and 42%) according to the applied criteria and the setting, with most systems classifying as MCI individuals with impairment in one or more cognitive domains (executive functions, memory, language, or visuospatial skills) and substantially normal functional activities (Stephan et al., 2007). The diagnosis of MCI was characterized for the first time in 1999 by the Petersen criteria and involves the classification into amnestic and non-amnesic subtypes (aMCI, naMCI, respectively). In amnestic MCI, memory is the dominant problem, while in non-amnestic types of MCI, cognitive impairment affects functions other than memory. It is therefore essential to adequately evaluate memory and other cognitive functions with specific tasks (Smirni et al., 2019).

Diagnosis of MCI in presence of subtle symptoms can be challenging. In these cases, it is necessary to document the cognitive decline from the patient history and to establish the presence of cognitive impairment by means of neuropsychological testing (Petersen, 2011). The National Institute on Aging and the Alzheimer’s Association remarked in 2011 that longitudinal evidence of progressive decline in cognition could support the diagnosis of MCI due to Alzheimer’s disease (AD) and could allow assessment of the potential benefits of early treatment (Albert et al., 2011).

A cognitive screening test is the first step in the detection of cognitive impairment in primary, secondary and tertiary care settings (Arevalo-Rodriguez et al., 2013). Short cognitive tests are very useful to detect individuals with cognitive impairments (Brown, 2015). The cognitive screening is generally based on a short cognitive evaluation with a paper-and-pencil modality, easy to administer, that takes no longer than 10 min. The final global score, obtained from the evaluation, is then used to determine which individuals need a more comprehensive neuropsychological assessment (these individuals are usually identified based on a global score cut-off value) (Boustani et al., 2003).

The most used screening cognitive test is the Mini Mental State Examination (MMSE) (Folstein et al., 1975). It has become the best-known and the most used short cognitive screening test for dementia in practice and research, both in clinical and community settings (Galasko et al., 1990; Mitchell, 2009; Nieuwenhuis-Mark, 2010). The MMSE is less useful to assess mild cognitive decline or psychiatric conditions (Naugle and Kawczak, 1989; O’Bryant et al., 2008).

In the clinical setting, another popular test is the Montreal Cognitive Assessment (MoCA). The MoCA was devised specifically with more difficult items to increase the sensitivity for MCI detection in the clinical setting, but also included frontal and executive function items to increase sensitivity for atypical dementia syndromes (Nasreddine et al., 2005; Bosco et al., 2017, 2020).

Other well-known brief cognitive rating scales, such as the Abbreviated Mental Test and Mental Status Questionnaire, suffer from problems of poor sensitivity and specificity and must be administered by expert and well trained personnel (MacKenzie et al., 1996).

The Test Your Memory (TYM) is a self-administered cognitive screening test requiring minimal supervision, which is validated for the screening of Alzheimer’s disease and other dementias (Brown J. et al., 2019). The TYM has been translated into several languages and it was used in different clinical settings (Hanyu et al., 2011; Hancock and Larner, 2011; Ojeda et al., 2011; van Schalkwyk et al., 2012; Szczesniak and Wojtynska, 2013; Muñoz-Neira et al., 2014; Postel-Vinay et al., 2014).

The National Institute for health and Care Excellence recommended, among several tools, the use of TYM test in non-specialist settings (National Institute for Health and Clinical Excellence, 2018).

The TYM was designed to meet three critical requirements: (1) take minimal operator time to be administered, (2) test a reasonable range of cognitive functions, (3) be sensitive in detecting mild AD (Brown et al., 2009). The TYM has been shown to have better performance in detecting AD compared with more traditional cognitive screening tests, such as the MMSE (Brown J. et al., 2019).

The aim of the present study was twofold: (1) to define the discrimination ability of the TYM Italian version (TYM-I) in detecting cognitive dysfunction in a clinical setting; (2) to describe which TYM-I subtests better discriminate between the amnestic and the non-amnestic cognitive dysfunction.

## Materials and Methods

### Participants

The patients included in the study were MCI consecutive patients attending to the Center for Neurodegenerative Diseases and the Aging Brain, University of Bari “Aldo Moro” at Pia Fondazione “Card. G. Panico” (Tricase, Lecce, Puglia, Italy), a tertiary center for dementia and other neurodegenerative diseases, between October 2013 and December 2014. The MCI diagnosis was based on the International Working Group on MCI criteria (Winblad et al., 2004). MCI were further subdivided in amnestic MCI (aMCI) if the memory domain was impaired, and non-amnestic MCI (naMCI) if the impairment was only in non-memory domains.

The healthy controls (HCs) were recruited from a collaborative network of general practitioners working in the Puglia Region, the same administrative region of the Center. The subjects, after invitation to participate in the study and obtaining a formal written consent, could decide to perform the test the same day or reschedule according to their schedule. The test was performed in a room, used exclusively for cognitive administration, at the presence of the neuropsychologist who provided help if requested, according to the TYM administration rules. Subjects were excluded, through the examination of general practitioner records, if history of major neurological or psychiatric disorders were ascertained.

In this study, we excluded patients with severe cognitive impairment due to dementia, according to neurological and neuropsychological assessment.

All subjects provided written informed consent before enrollment. This validation study was approved by our local institutional review board (ASL Lecce and Bari ethical committees).

### Cognitive Assessment

MCI patients’ cognitive profile was defined based on the following neuropsychological battery of tests: (1) Mini Mental State Examination (MMSE) (Folstein et al., 1975), Frontal Assessment Battery (FAB) (Dubois et al., 2000) to assess the global cognitive functioning; (2) Rey Auditory Verbal Learning Test (Caltagirone et al., 1995) to evaluate the episodic memory; (3) the Symbol Digit Modalities Test-oral version (Nocentini et al., 2006), the Stroop Test (Caffarra et al., 2002) and the Verbal Fluency Test (Caltagirone et al., 1995) to examine the executive functions; (4) Semantic fluency test (Capasso and Miceli, 2001) and the Boston Naming Test-short version (Kaplan et al., 1978) to assess the language; (5) the Clock Drawing Test (Caffarra et al., 2011) to evaluate the visuo-constructive abilities. Mood state was evaluated with the 30-items Geriatric Depression Scale (GDS) (Yesavage et al., 1982-1983) and The Apathy Evaluation Scale (SAS) (Starkstein et al., 2006).

### TYM Test

The self-administered TYM screening test is a series of 10 tasks on a double-sided sheet of card with spaces for the patient to fill in responses (Brown et al., 2009). No sub-items are timed and there is no time limit.

The cognitive domains are the following:

- Place and person orientation (10 points): the subject must write his full name, current date, month and year and his date of birth;
- Ability to copy (two points): the sentence to copy is “Good citizens always wear stout shoes”;
- Retrograde memory (semantic knowledge) (three points): the subject is required to write the name and surname of the prime minister and the date of First World War;
- Calculation (four points): four sums are required;
- Verbal fluency (four points): the subject must list four creatures beginning with the letter “s”;
- Similarities (four points): the subject must write how a carrot is like to a potato and a lion like a wolf;
- Naming (five points): the subject must write the names of jacket parts;
- Visuospatial abilities (seven points): circles and squares are present on the sheet. The circles are arranged to form the letter “W.” the subject is required to join only the circles to form a letter;
- Anterograde memory (six points): this task requires the recall of a copied sentence.

The ability to do the test is also scored from five points for patients requiring no help to zero point for patients requiring major help. Help is limited to encouragements and aims at controlling the adequacy of the performance. Specifically, the help consists in repeating the instructions of the sub-items if the subject requests it, without any facilitation in the answer. Furthermore, the help consists in indicating to the subject any sub-tests that have remained incomplete or omitted.

The help scores are categorized in 4 categories: any help (score equal to 5), minor help (score equal to 3 or 4), moderate help (score equal to 1 or 2), complete help (score equal to 0). The overall TYM global score ranges from 0 to 50 points (Brown J. et al., 2019).

The authors received written permission to translate and validate the TYM test in Italian from the author, Dr. Brown.

The Italian version of the TYM (TYM-I) was obtained modifying the original version in order to be consistent and equivalent to the English version, taking into account linguistic and cultural factors.

Specifically, two sections of the original test, Semantic Knowledge and Visuospatial abilities, were modified to improve their comprehension and cultural adequacy as follows:

1. the words “prime minister” were substituted with “head of government,”
2. the date of the First World War is relative to the year of entry of Italy,
3. the letter “W” is not included in the original Italian alphabet. Therefore, in the visuospatial abilities section this letter was replaced with the letter “M.”

All the changes were approved by the original author of the test.

All recruited subjects underwent TYM-I test.

### Statistical Analysis

Patients’ characteristics are reported as mean and standard deviation (SD), and frequencies and percentages, for continuous and categorical variables, respectively. Spearman’s rank correlation coefficient was used to assess associations between quantitative variables. The reliability and the internal consistency of the TYM-I has been measured using Cronbach’s alpha coefficient.

Comparisons between MCI patients and HCs variables’ distributions were conducted using Mann–Whitney U test for quantitative variables and Pearson’s chi-squared for categorical ones. Differences between aMCI, naMCI, and HCs groups were assessed using Pearson χ^2^ for categorical variables and Kruskal–Wallis followed by Nemenyi *post hoc* test for quantitative ones. The Receiver Operating Characteristic (ROC) curve for TYM-I global score was used to detect the optimal cut-off value in the discrimination between HCs and MCI patients, considering both sensitivity and specificity jointly. Specifically, the cut-off value maximizing the Youden’s index was selected as “optimal.” The Area Under the Curve (AUC) was computed for both MMSE and TYM-I global scores in order to assess and compare discrimination abilities.

The AUC, sensitivity, specificity, positive likelihood ratio and negative likelihood ratio were calculated along with their 95% bootstrapped confidence interval (CI), considering 2,000 stratified sampling replications. ROC analysis was performed overall and separately for each combination of age group (40–60, 61–70, or 71–90 years) and educational level (0–8 or 9–18 years).

We also reported the regression coefficients needed to allow age-education correction based on normative data for the TYM-I. Specifically, we reported the intercept and the regression coefficients of the linear regression model with the TYM-I global score as dependent variable and age and the square root of education years as independent variables, estimated using HCs data.

Finally, we computed the percentage of individuals with the minimum and maximum theoretical score in the two groups in order to assess the presence of floor (lowest score) and ceiling (highest score) effects for TYM-I. All analyses were performed using R (v 3.3.1) and R studio (v 1.0.153). A *p*-value lower than 0.05 was considered as statistically significant.

## Results

We enrolled 94 MCI patients (*n* = 40 aMCI; *n* = 54 naMCI) and 134 HCs.

Table 1 shows the sociodemographic characteristics and clinical profiles for both MCI patients and healthy controls. There were significant differences in age and education between the groups. The MCI group was significantly older than the HCs group (*p* < 0.0001) and had significantly lower education (*p* = 0.0004) compared to HCs. The MCI group had statistically significant lower scores than HCs on MMSE (*p* < 0.0001), FAB (*p* < 0.0001) and higher GDS scores (*p* = 0.0137). The naMCI patients were younger (*p* = 0.0182) and with better performance on MMSE (*p* = 0.0029) compared to aMCI ones. The comparative analyses among different groups on TYM-I subscores are showed in Table 2. Statistically significant differences were found between HCs and MCI group on TYM-I global score and on 10 out of 11 subscores. The aMCI group performed worse on orientation (*p* < 0.0001), copying (*p* = 0.0098), visuospatial 2 (*p* = 0.0179), anterograde memory (*p* = 0.0010), help (*p* = 0.0426) subscores than naMCI. A total of 14 (35.00%) aMCI patients did not require any help, 11 (27.50%) required moderate help, 15 (37.50%) needed minor help. No aMCI patient needed complete help. In the naMCI group 18 (33.33%) patients required minor help, 4 (7.41%) moderate help, 2 (3.70%) complete help, 30 (55.56%) any help. Cronbach α was 0.78, showing good internal consistency of the TYM-I. The TYM-I global score correlated with age (*p* < 0.0001; *r* = -0.429), years of education (*p* < 0.0001; *r* = 0.410), MMSE (*p* < 0.0001; *r* = 0.631), FAB (*p* < 0.0001; *r* = 0.616), and GDS (*p* = 0.0211; *r* = -0.155) scores.

**TABLE 1:** Comparison of sociodemographic, clinical, and cognitive variables between MCI and HCs groups.

	MCI *n* = 94	HCs *n* = 134	*p*-value MCI vs. HCs	aMCI *n* = 40	naMCI *n* = 54	*p*-value aMCI vs. HCs	*p*-value naMCI vs. HCs	*p*-value aMCI vs. naMCI
**Age, years**								
Media ± *SD*	70.5 ± 9.21	64.2 ± 8.23	**< 0.0001**	73.88 ± 7.85	68 ± 9.41	**< 0.0001**	**0.0161**	**0.0182**
Median	72	64.5		76	69.5			
(Range)	(43–87)	(42–83)		(59–85)	(43–87)			
**Sex**								
Male number (%)	42 (44.68%)	61 (45.52%)	1.0000	21 (52.5%)	21 (38.89%)			
Female number (%)	52 (55.32%)	73 (54.48%)		19 (47.5%)	33 (61.11%)			
**Education, years**								
Media ± *SD*	7.85 ± 4.72	9.78 ± 4.13	**0.0004**	7.58 ± 4.94	8.06 ± 4.59	**0.0169**	**0.0240**	0.9384
Median	5	8		6.5	5			
(Range)	(0–18)	(3–17)		(0–18)	(2–18)			
**MMSE**								
Media ± *SD*	26.28 ± 3.11	29.19 ± 1.1	**< 0.0001**	24.93 ± 3.18	27.28 ± 2.66	**< 0.0001**	**< 0.0001**	**0.0029**
Median	27	30		25	28			
(Range)	(17–30)	(25–30)		(17–30)	(17–30)			
**FAB**								
Media ± *SD*	12.8 ± 2.94	16.01 ± 2.01	**< 0.0001**	12.4 ± 2.88	13.09 ± 2.98	**< 0.0001**	**< 0.0001**	0.5587
Median	13	16		13	13			
(Range)	(5–18)	(6–18)		(6–17)	(5–18)			
**GDS**								
Media ± *SD*	9.91 ± 6.42	7.8 ± 5.48	**0.0137**	9.29 ± 5.99	10.37 ± 6.74	0.3642	0.0562	0.8274
Median	9	7		8	10			
(Range)	(0–27)	(0–26)		(0–25)	(1–27)			
**SAS**								
Media ± *SD*	11.16 ± 7.72	9.37 ± 6.17	0.1087	11.47 ± 7.68	10.96 ± 7.82			
Median	11	8		11	10			
(Range)	(0–36)	(0–34)		(0–30)	(0–36)			

*When a statistically significant difference between aMCIs, naMCIs, and HCs groups was found, post hoc analyses were also conducted (last three columns). Significant p-values are bolded for emphasis.*

**TABLE 2:** Comparison of performance on TyM-I between MCI and HCs groups.

TyM-I Subscore (maximum)	MCI *n* = 94	HCs *n* = 134	*p*-value MCI vs. HCs	aMCI *n* = 40	naMCI *n* = 54	*p*-value aMCI vs. HCs	*p*-value naMCI vs. HCs	*p*-value aMCI vs. naMCI
Orientation (10)	8.97 ± 1.69	9.85 ± 0.48	**< 0.0001**	8.18 ± 2.06	9.56 ± 1.04	**< 0.0001**	0.2097	**< 0.0001**
Copying (2)	1.68 ± 0.66	1.96 ± 0.24	**< 0.0001**	1.5 ± 0.78	1.81 ± 0.52	**< 0.0001**	0.1833	**0.0098**
Retrograde Memory (3)	0.97 ± 0.97	1.37 ± 0.88	**0.0011**	0.7 ± 0.85	1.17 ± 1.00	**0.0003**	0.3744	0.0552
Calculation (4)	3.17 ± 1.14	3.5 ± 0.71	0.1131	3.08 ± 1.19	3.24 ± 1.11			
Phonemic fluency (4)	2.03 ± 1.39	2.96 ± 1.07	**< 0.0001**	1.8 ± 1.42	2.2 ± 1.35	**< 0.0001**	**0.0024**	0.4187
Similarities (4)	1.83 ± 1.29	2.82 ± 1.19	**< 0.0001**	1.52 ± 1.2	2.06 ± 1.32	**< 0.0001**	**0.0014**	0.1862
Naming (5)	4.23 ± 1.15	4.73 ± 0.65	**< 0.0001**	4.05 ± 1.3	4.37 ± 1.01	**0.0019**	**0.0227**	0.6491
Visuospatial 1 (3)	1.48 ± 1.1	2.23 ± 0.95	**< 0.0001**	1.3 ± 1.14	1.61 ± 1.07	**< 0.0001**	**0.0018**	0.4598
Visuospatial 2 (4)	2.91 ± 1.4	3.59 ± 0.96	**< 0.0001**	2.5 ± 1.47	3.22 ± 1.27	**< 0.0001**	0.1658	**0.0179**
Anterograde memory (6)	1.94 ± 1.98	4.04 ± 1.87	**< 0.0001**	0.95 ± 1.54	2.67 ± 1.97	**< 0.0001**	**0.0004**	**0.0010**
Help (5)	3.84 ± 1.45	4.85 ± 0.42	**< 0.0001**	3.48 ± 1.5	4.11 ± 1.36	**< 0.0001**	**<0.0001**	**0.0426**
Global score	33.05 ± 7.86	41.9 ± 4.6	**< 0.0001**	29.05 ± 8	36.02 ± 6.35	**< 0.0001**	**<0.0001**	**0.0053**

*Significant p-values are bolded for emphasis.*

**FIGURE 1:**
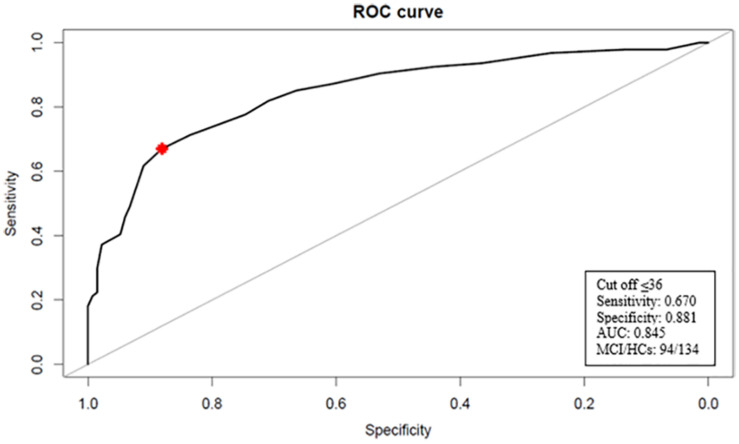
Receiver operating characteristic (ROC) curve for TYM-I. The optimal cut-off value for discriminating between HCs and MCI patients is indicated with a star. AUC, area under the curve; MCl, mild cognitive impairment; HCs, health controls.

**FIGURE 2:**
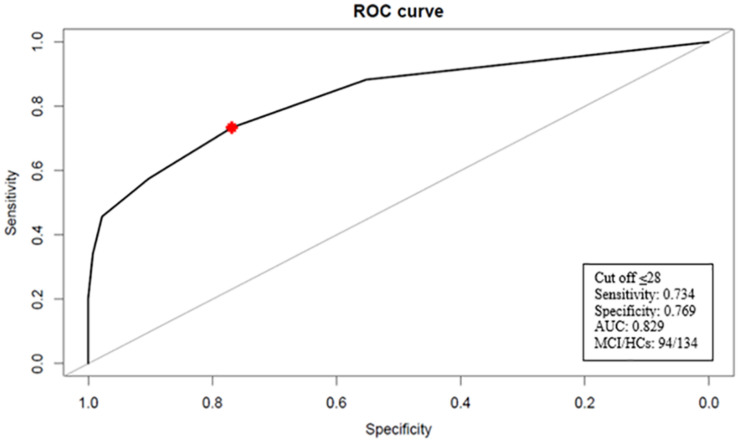
Receiver operating characteristic (ROC) curve for MMSE. The optimal cut-off value for discriminating between HCs and MCI patients is indicated with a star. AUC, area under the curve; MCI, mild cognitive impairment; HCs, health controls.

Results of the ROC curve analyses for the TYM-I and MMSE are displayed in Figures 1, 2. It should be noticed that TYM-I discrimination ability in distinguishing between HCs and MCI group was similar (AUC = 0.845; 95% CI: 0.789–0.895) (Figure 1) to the one of MMSE (AUC = 0.829; 95% CI: 0.773–0.880) (Figure 2). A TYM-I global score lower or equal to 36 was found to be the optimal cut-off in detecting MCI with a sensitivity of 67.02% (95% CI: 57.45–76.60) and a specificity of 88.06% (95% CI: 82.09–93.28).

Results from ROC curves for different classes of age (40–60; 61–70; 71–90) and educational level (low 0–8; high 9–18) are reported in Table 3. The TYM-I showed the highest discrimination ability in subjects aged 71 and over with high educational level (AUC = 0.89; 95% CI: 0.72–1.00).

**TABLE 3:** Discrimination ability of TyM-I in detecting MCI for different age and educational levels.

Age	Education	HCs/MCI	Cut off	Specificity (95% CI)	Sensitivity (95% CI)	AUC (95% CI)	LR+ (95% CI)	LR- (95% CI)
**41–60**	**9–18**	22/6	≤ 41	0.86 (0.73–1.00)	0.50 (0.17–0.83)	0.61 (0.29–0.89)	3.67 (0.73–Inf)	0.58 (0.17–1.05)
**41–60**	**0–8**	20/10	≤ 39	0.75 (0.55–0.90)	0.80 (0.50–1.0)	0.77 (0.58–0.92)	3.20 (1.60–10.05)	0.27 (0.00–0.67)
**61–70**	**9–18**	29/8	≤ 36	1.00 (1.00–1.00)	0.88 (0.63–1.00)	0.88 (0.64–1.00)	Inf (Inf–Inf)	0.13 (0.00–0.38)
**61–70**	**0–8**	34/15	≤ 35	0.88 (0.76–0.97)	0.53 (0.27–0.80)	0.73 (0.57–0.88)	4.53 (1.76–22.67)	0.53 (0.23–0.84)
**71–90**	**9–18**	11/15	≤ 40	0.82 (0.55–1.00)	1.00 (1.00–1.00)	0.89 (0.72–1.00)	5.50 (2.20–Inf)	0.00 (0.00–0.00)
**71–90**	**0–8**	18/40	≤ 36	0.83 (0.67–1.00)	0.85 (0.73–0.95)	0.87 (0.75–0.96)	5.10 (2.47–Inf)	0.18 (0.06–0.32)

*Sensitivity, Specificity, Positive likelihood ratio (LR+), Negative likelihood ratio (LR-) and corresponding 95% confidence intervals are also reported. Age and education are bolded for emphasis.*

The intercept of the linear regression model of the TYM-I global score among the HCs was 41.4306 (95% CI: 34.7089–48.1524), while the regression coefficients for age and the square root of education years were -0.1094 (95% CI: -0.1972 to -0.0216) and 2.4550 (95% CI: 1.3839–3.5261), respectively.

A MMSE score lower or equal to 28 was found to be the optimal cut-off in detecting MCI with a sensitivity of 73.40% and a specificity of 76.87% in our sample.

The 6.71% (*n* = 9) of healthy controls scored 48–49 in the TYM-I. The highest TYM-I score observed in the MCI group was 48 (2.13%, *n* = 2). No individual reached the maximum global score.

## Discussion

Cognitive dysfunction is a common condition among the elderly. MCI can be considered as an intermediate status along a continuum from normal cognition and subjective cognitive complaint to dementia (Petersen et al., 1999). However, MCI is not always precursor of dementia, MCI can remain stable or could even revert to normal cognitive function over time. Detection of MCI is important for prevention because intervention on reversible risk factors may slow or halt progression to dementia (Sanford, 2017). Rates of progression to dementia are very heterogeneous. For example, the amnestic MCI rates of progression to Alzheimer’s disease, range from 5 to 16% per year (Tierney et al., 1996; Artero et al., 2003).

The TYM is a brief screening tool to detect cognitive dysfunction. Several validation studies were conducted to assess the TYM performances in detecting Alzheimer’s disease patients across different regions and clinical settings, such as memory clinic and primary care (Hanyu et al., 2011; van Schalkwyk et al., 2012; Szczesniak and Wojtynska, 2013; Muñoz-Neira et al., 2014; Iatraki et al., 2017; Brown J. et al., 2019).

In our study, the best TYM-I sensitivity and specificity in discriminating between MCI patients and HCs were obtained with a global score lower than or equal to 36. However, in this assessment we defined the optimal cut-off assuming an equal weight for the proportion of false positive and false negative errors. In according with other studies on MMSE cut off in MCI, in our sample the best cut-off value for the MMSE was 28 (Ciesielska et al., 2016). Our results confirmed that the accuracy of these two screening tests in detecting cognitive dysfunction is similar (Hanyu et al., 2011; Hancock and Larner, 2011; Koekkoek et al., 2013; Brown J. et al., 2019).

The mean TYM-I scores in our HC and MCI samples were lower than those in the control and MCI samples evaluated in Brown’s et al. original studies (Brown et al., 2009; Brown J. et al., 2019). However, results are inconsistent among investigations from different countries and several studies reported average scores in line with those of the present study in both controls (Hancock and Larner, 2011; Abd-Al-Atty et al., 2012; van Schalkwyk et al., 2012; Muñoz-Neira et al., 2014; Postel-Vinay et al., 2014; Iatraki et al., 2014, 2017; Kolozsvári et al., 2017; van de Zande et al., 2017; Li et al., 2018) and MCI patients (Hancock and Larner, 2011; Muñoz-Neira et al., 2014; van de Zande et al., 2017). Whether this heterogeneity might be partly attributed to a difference in educational levels should be accurately addressed in future studies.

Considering the demographic differences between the MCI and HCs groups, we have reported age-education corrections. We both presented age-education specific cut-offs for the TYM-I based on ROC analysis, and regression coefficients for age-education adjustment obtained modeling with linear regression the normative data. However, it is important to underscore that the correction for age and education for cognitive tests has been strongly criticized because of its negative impact on the test discrimination ability (Kramer et al., 1998). The rationale that supports this theory shows that age and education are associated both to the test score and to the probability of diagnosis. The age and education correction makes the group of diseased and non-diseased artificially similar ignoring the association between these factors and cognitive impairment and therefore decreasing the discrimination ability (O’Connell et al., 2004).

The TYM-I global score was worse in aMCI patients compared to naMCI ones. The subscores analysis showed that aMCI patients had worse performances on orientation, visuospatial abilities and anterograde memory and needed a significant percentage of mild/moderate help during TYM-I administration. These results suggested that in a cognitive screening phase a greater impairment in these subtests could be indicative of the presence of amnestic dysfunction. These considerations are important if we consider that a subject with an amnestic cognitive dysfunction has a minor clinical symptom and could later develop dementia due to Alzheimer’s disease (Albert et al., 2011; Silva et al., 2013).

In our sample the TYM-I did not show a ceiling effect and this perhaps makes it more suitable to detect cognitive differences in subjects with high cognitive performance.

We recognize some limitations in our work. First, we collected a relatively small sample size, especially for the ROC analyses in strata of age and education. Second, our study design is not considered the best design for validating diagnostic tools because the diagnostic test is evaluated in a group of patients already known to have the diagnosis of interest and a separate group of “normal” subjects (Lijmer, 1999). However, this design is preferable to define thresholds for neuropsychological screening tests compared to the ones in which only normative data are collected. Third, in our study we compare MMSE and TYM-I, which are scales with different range of assessment. The two tests, even if show similar ability in discriminating MCI from HCs, probably explore different cognitive functions (TYM-I describes executive functions that are not investigated in MMSE). TYM-I and MMSE identify MCI in a possible different stage of the diseases, as shown from the comparison of amnestic and non-amnestic MCI.

A significant strength of our study compared to others is the presence, of a full neuropsychological evaluation to define the cognitive profile for all MCI patients. This careful and extensive neuropsychological examination is missing in the most of previous studies on the same topic. Another strength is the recruitment of HCs in GPs settings. The GPs setting for the recruitment of HC is particularly suited to be representative of the population and allows to evaluate the cognitive disorders in its entire spectrum.

## Conclusion

In conclusion, the TYM-I is a screening tool that could be increasingly used in Italy in clinical setting because of its good discrimination ability in detecting MCI. This tool could be also useful in primary care setting and population-based studies. In clinical setting of tertiary and research centers, the use of TYM-I should be followed by the administration of an extended neuropsychological battery to define the cognitive profile and severity with optimal accuracy.

## Data Availability Statement

The raw data supporting the conclusions of this article will be made available by the authors, without undue reservation, to any qualified researcher.

## Ethics Statement

The studies involving human participants were reviewed and approved by ASL Lecce and Bari ethical committees. The patients/participants provided their written informed consent to participate in this study.

## Author Contributions

MB: study concept and design, acquisition of data. MP: analysis and interpretation. AB, RC, and RT: critical revision of the manuscript for important intellectual content. CM and CD: acquisition of data. UL: critical revision of the manuscript for important intellectual content and study supervision. GL: funding, study concept, design, and supervision. All authors contributed to the article and approved the submitted version.

## Conflict of Interest

The authors declare that the research was conducted in the absence of any commercial or financial relationships that could be construed as a potential conflict of interest. The reviewer AL declared a shared affiliation, with no collaboration, with one of the authors GL to the handling editor at the time of the review.
